# The Complete Chloroplast Genome Sequence of *Machilus chuanchienensis* (Lauraceae): Genome Structure and Phylogenetic Analysis

**DOI:** 10.3390/genes13122402

**Published:** 2022-12-18

**Authors:** Xue Bai, Juan Peng, Yongyi Yang, Biao Xiong

**Affiliations:** College of Tea Science, Guizhou University, Guiyang 550000, China

**Keywords:** *Machilus chuanchienensis* S. Lee, comparative plastid genomics, Illumina sequencing, genome skimming

## Abstract

*Machilus chuanchienensis* is an ecological tree distributed in southwestern China. It has a significant valuation with making Hawk tea using its leaves, an ethnic traditional tea-like beverage with a long history in Chinese tea culture. The whole chloroplast (cp) genome is an ideal model for the phylogenetic study of Lauraceae because of its simple structure and highly conserved features. There have been numerous reports of complete cp genome sequences in Lauraceae, but little is known about *M. chuanchienensis*. Here, the next-generation sequencing (NGS) was used to sequence the *M. chuanchienensis* cp genome. Then, a comprehensive comparative genome analysis was performed. The results revealed that the *M. chuanchienensis*’s cp genome measured 152,748 base pairs (bp) with a GC content of 39.15% and coded 126 genes annotated, including comprising eight ribosomal RNA (rRNA), 36 transporter RNA (tRNA), and 82 protein-coding genes. In addition, the cp genome presented a typical quadripartite structure comprising a large single-copy (LSC; 93,811) region, a small single-copy (SSC; 18,803) region, and the inverted repeats (IRs; 20,067) region and contained 92 simple sequence repeat (SSR) locus in total. Phylogenetic relationships of 37 species indicated that *M. chuanchienensis* was a sister to *M. balansae*, *M. melanophylla*, and *M. minutiflora*. Further research on this crucial species may benefit significantly from these findings.

## 1. Introduction

*Machilus chuanchienensis* S. Lee, an indeciduate tree belonging to the Lauraceae family, is distributed in low-altitude montane forests in southeastern China. It contains substances such as terpenoids and flavonoids, quercitrin, kaempferol, hyperin, astragalin, isoquercitrin, and quercetin [1]. As a tea-like plant, *M. chuanchienensis* can be utilized to make a traditional folk beverage Hawk tea [2], one of the non-Camellia teas, which has yellowish-red tea soup with camphor-aromatic smell [3]. According to *the Compendium of Materia Medica*, a classic book of traditional Chinese medicine, *M. chuanchienensis* can be used as Chinese medicine because of rich in polyphenols, flavonoids, vitamins, minerals, and other compounds and is free from caffeine. Moreover, due to the pharmacological effects of Hawk tea, such as antidiabetic [4], acting antioxidant, hypolipidemic, and anti-inflammatory properties [5], consumers sincerely like this beverage [6], and in some places, its consumption is even higher than that of green tea [7].

Lauraceae species are extensively spread in the world’s tropical and subtropical regions, which contain about 2500–3000 species from around 50 genera [8]. Lauraceae is an evolutionarily complex and taxonomically controversial group in which the phylogenetic location in the disputed genera (such as *Actinodaphne* Nees and *Sassafras* J. Presl) has been controversial [9,10]. Although *Litsea coreana* var. *lanuginose* is one of the most researched primary raw materials for making Hawk tea [11], there is a lack of relevant research on other plants belonging to Lauraceae species [12], especially for *Machilus chuanchienensis*, making it challenging to explore Hawk tea germplasm resources and study the phylogenetic relationships of its material-species.

As important plastids in plant cells, chloroplasts are involved in the main functions, including photosynthesis [13], carbon sequestration, starch storage, nitrogen metabolism, fatty acids, and nucleic acid synthesis [14]. In addition, they self-replicate organelles with their DNA and RNA and have relatively independent genetic replication mechanisms like mitochondria [15]. Although the structure of the cp genome is remarkably conserved [16], its size and gene content have polymorphism related to genes reorganization and IRs region growth and contraction [17], which has been used for molecular identification, population genetics, endangered species conservation, and phylogenetic analysis [18,19]. High-throughput technologies’ successful development has made the sequencing of chloroplast genomes economical and efficient, significantly developing chloroplast-based related studies. One of the most valuable methods at the moment is building a phylogenetic tree of the Lauraceae utilizing the cp genomes, provided that the technology for sequencing and assembling nuclear and mitochondrial genomes of Lauraceae is not to be universal [20]. However, using whole genome sequencing (WGS) technologies to research *M. chuanchienensis*’ genome has yet to be reported, and its taxonomic position within Lauraceae has not been precisely placed.

In this study, we first assembled the whole cp genome sequence of *M. chuanchienensis* using NGS technology. In addition, a comprehensive analysis of the whole cp genomic features was performed. Then, a phylogenetic tree was constructed using the cp genome sequences to explore the phylogenetic relationship between *M. chuanchienensis* and other species, which provided some theoretical basis for understanding the genomic features of *M. chuanchienensis* and its phylogenetic relationships.

## 2. Materials and Methods

### 2.1. Experimental Materials and DNA Extraction

Ya’an city, Sichuan Province, China’s habitats were the locations where the samples were taken (103°00′ E, 29°98′ N), and the fresh leaves were picked in the form of one bud and two leaves. The voucher specimen was stored at the Herbarium of Forestry College, Guizhou University. The accession number is YA202108MC02. The genomic DNA of *M. chuanchienensis* was extracted from healthy fresh young leaves by modified CTAB method [21] and was detected by ultraviolet spectrophotometer and 1% agarose gel electrophoresis [22].

### 2.2. DNA Sequencing, Genome Assembly and Annotations

Genomic DNA qualified for library construction that constructs a pair-end library with an insertion size of 150 bp was sequenced using the Illumina NovaSeq 6000 platform in NGS. Sequencing data was acquired for quality control, and then the GetOrganelle script was run on a Linux system to assemble the genome [23]. Bandage software was used to visualize if it is a circle [24]. The *Neolitsea homilantha* cp genome sequence was used as a reference to annotate genome using the cp genome annotation website CPGAVAS2 and the annotation results provided collinear analysis, gene function classification information, and intron information tables [25]. The tRNA was then identified by the tRNAscan-SE 1.21 program [26], and a circle diagram of the cp genome was drawn using the OrganellarGenomeDRAW v.1.3.1 [27].

### 2.3. Simple Sequence Repeats (SSRs) and Repeat Sequences Analysis

SSRs in the cp genome of *M. chuanchienensis* were identified by the microsatellite identification online tool (MISA) [28]. The search parameters for a minimum number of repeats were set as follows: 10 for mononucleotide repeats, five for dinucleotide repeats, four for trinucleotide repeats, and three for tetra-, penta-, and hexanucleotide repeats. In addition, ‘100 bp’ was selected as the minimum distance between the two SSRs. The two SSRs were considered to be a compound microsatellites if the distance was less than 100 bp. REPuter [29,30] was used to identify repeat sequences with the parameters reported [31,32,33].

### 2.4. Putative RNA Editing Site and Codon Usage

The coding sequences (CDS) were obtained by the CPGAVAS2 online software to anticipate the potential RNA editing sites in the *M. chuanchienensis* chloroplast genome. Then, they were submitted to the predictive RNA editors for the plant chloroplast (PREP-cp) database [34]. The *M. chuanchienensis*’s relative synonymous codon usage (RCSU) and codon usage count were examined using MEGA X [35].

### 2.5. Genomic Comparison with Other Species in Machilus

The *M. chuanchienensis* cp genome and four related species were homogeneously compared using the mVISTA program [36] made a homogeneity comparison. Of the *M. chuanchienensis* cp genome and the four related species, including *M. balansae*, *M. grijsii*, *M. robusta*, and *M. yunnanensis*, all of which were in the genus *Machilus*. Among them, the *M. chuanchienensis* cp genome was selected as the reference with the Shuffle-LAGAN.

The PhyloSuite software [37] extracted 76 common protein-coding genes from *M. balansae*, *M. grijsii*, *M. chuanchienensis*, *M. robusta*, and *M. yunnanensis*. The extracted gene sequences were initially aligned using the MAFFT software [38,39]. Then, the ratios of nonsynonymous (Ka) to synonymous (Ks) substitutions (Ka/Ks) were calculated by the DnaSP software [40]. The expansion and contraction of IR boundaries were detected using the web program IRscope [41]. Analyzing the nucleotide diversity (Pi) value also utilized the DnaSP program [40]. 500 bp was chosen as the step size and window length.

### 2.6. Phylogenetic Analysis

To create a maximum likelihood (ML) phylogenetic tree, we retrieved the cp genome sequences of 34 species from the Lauraceae family and two outgroup species from the Calycanthaceae, including *Chimonanthus nitens* (NC_042745) and *Idiospermum australiense* (NC_042743). Eighty-one CDS_NCU genes were extracted, aligned, and merged using PhyloSuit software [37]. The ML phylogenetic tree was conducted by IQ-TREE version 2 [42], with a TVM + F + I + G4 model chosen based on the Bayesian Information Criterion [43] from the result of ModelFinder.

## 3. Results

### 3.1. Structure and Characteristics of the M. chuanchienensis Chloroplast Genome

**Figure 1:**
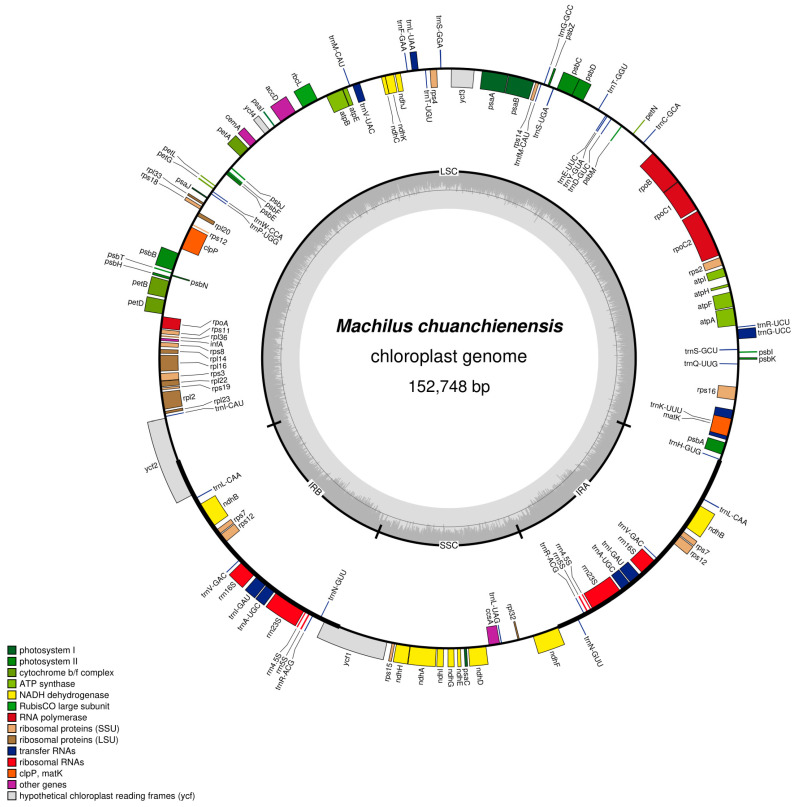
Chloroplast genome map of *M. chuanchienensis*. Genes on the outside of the main circle are transcribed clockwise, while genes on the inside are transcribed counterclockwise. The Organellar Genome Draw (OGDraw) online software was used to draw this map. Genes with different functions are represented by different colors. The gray portion of the inner circle indicates the GC content of the chloroplast genome.

**Figure 2:**
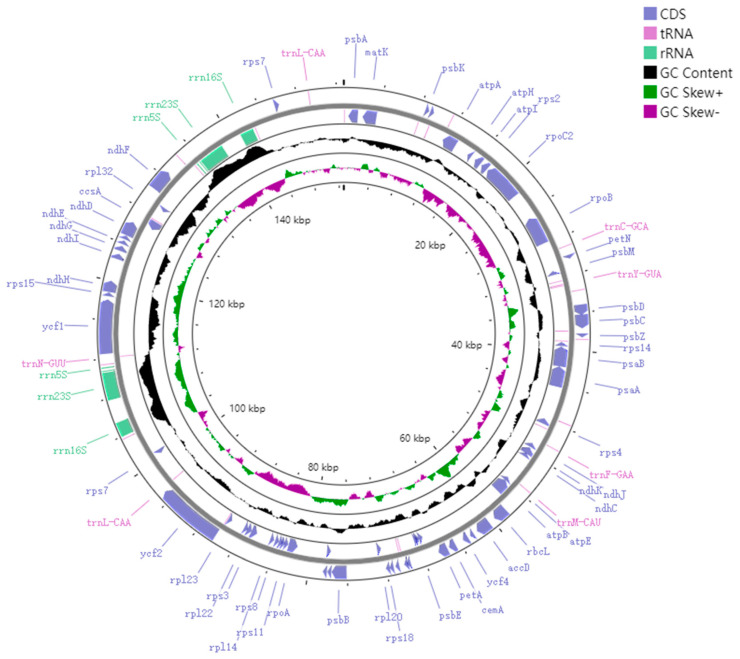
GC content of the *M. chuanchienensis*. This map was created using the web program GCView Server. The black portion represents changes in GC content in different regions of the genome. The deviation of G and C content in each single strand is called GC skew. The specific calculation method is (nG − nC)/(nG + nC), so GC skew + (green portion) means that the content of G is greater than that of C, and GC skew − (magenta portion) means that the content of G is less than that of C.

The *M. chuanchienensis* cp genome, like that of most angiosperms, is a covalently closed double-stranded cyclic molecule with a total length of 152,748 bp, including a small single-copy (SSC) region (18,803 bp), a large single-copy (LSC) region (93,811 bp) and a pair of inverted repeats (IRs) regions (20,067 bp) (Figure 1). The total GC content of the chloroplast genome was 39.15%, and the AT content was 60.85%, which had evident AT bias. In addition, there were some differences in the GC content for the IR, LSC, and SSC regions. The IR region had the highest GC content (44.43%), followed by the LSC region (37.94%) and the SSC region (33.92%) because the IR region’s rRNA genes have a high GC content. (Figure 2).

The chloroplast genome annotation results showed that *M. chuanchienensis* contained 126 functional genes, of which 82, 8, and 36, respectively, were protein-coding, rRNA, and tRNA genes. Of these genes, six tRNA genes (*trnA*-*UGC*, *trnL*-*CAA*, *trnI*-*GAU*, *trnR*-*ACG*, *trnV*-*GAC*, *trnN*-*GUU*), three protein-coding genes (*rps7*, *rps12*, *ndhB*), and four rRNA genes (*rrn5*, *rrn23*, *rrn4.5*, *rrn16*) were located in the IR region, all of which were duplicated once in the IRs regions (Table 1). In addition, the *rps12* gene had a trans-spliced structure, with its 5′ end in the LSC region and its 3′ end in the IR region.

Introns contribute significantly to the regulation of gene expression. There are 18 intron-containing genes in the *M. chuanchienensis* cp genome, including six tRNA (*trnG*-*UCC*, *trnI*-*GAU*, *trnA*-*UGC*, *trnK*-*UUU*, *trnL*-*UAA*, *trnV*-*UAC)* and 12 protein-coding genes (*rps12*, *ycf3, rps16*, *rpl16*, *petB*, *rpl2*, *petD*, *atpF*, *clpP*, *ndhA*, *rpoC1*, *ndhB*). The *clpP*, *rps12*, and *ycf3* contain two introns, and the others contain one intron (Table 2). The t*rnK*-*UUU* gene contains the protein-coded gene *matK* and the maximum intron with a length of 2507 bp, which has similar properties to other green plants [44].

### 3.2. Analysis of SSRs and Long Repeats

SSRs analysis showed that there were 92 SSRs loci, including 67 mononucleotides, ten dinucleotides, three trinucleotides, ten tetranucleotides, one pentanucleotide, and one hexanucleotide repeats. Mononucleotide SSRs were the most abundant, accounting for 72.83%. Moreover, A/T, AT/AT, and AAAT/ATTT motifs were 80.43%, indicating that SSRs of *M. chuanchienensis* preferred to use A and T bases (Table S1). Except for SSRs, a repeat (≥30 bp is considered a long repeat sequence. The *M. chuanchienensis* chloroplast genome had 31 long repeats in total, including 13 forward, 5 reverses, 2 complement, and 11 palindrome repeats (Table S2). The size of the repeats ranged from 30 to 72 bp, of which the longest repeat resided in the LSC region (72 bp).

### 3.3. Codon Usage and Putative RNA Editing Site within M. chuanchienensis

**Figure 3:**
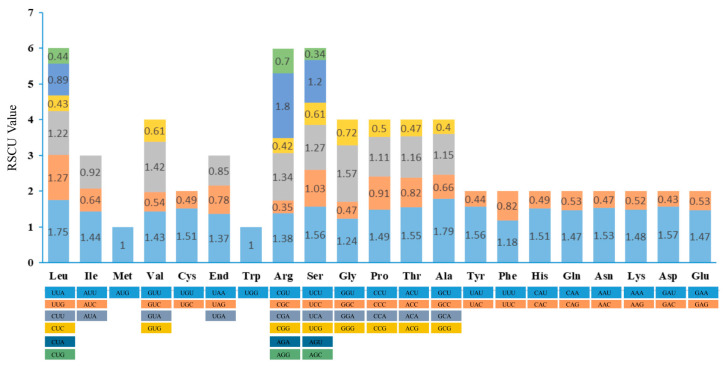
RSCU value of 20 amino acids and stop codons in all protein-coding genes of *M. chuanchienensis* chloroplast genome.

The *M. chuanchienensis* cp genome had 23,598 codons in its all protein-coding genes. (Table S3). Among these codons, the three most numerous amino acids were leucine (2396, 10.15%), isoleucine (2003, 8.49%), and serine (1847, 7.83%), while the three least numerous amino acids were cysteine (272, 1.15%), tryptophan (405, 1.72%), and methionine (557, 2.36%). Based on the calculation of relative synonymous codon use (RSCU), 31 codons with an A or T ending had RSCU >1 except TTG (Leucine, 1.27) and TCC (Serine, 1.03), and 31 codons had RSCU < 1, the vast majority of which ended in C or G, with only CTA (Leucine, 0.89) and ATA (Isoleucine, 0.92) ending in A. In addition, ATG and TGG had no codon bias (RSCU = 1) (Figure 3).

Prediction of putative RNA editing sites for *M. chuanchienensis* cp genes revealed a total of 123 editing sites, all with expected C-U transitions (Table S4). Of the 82 protein-coding genes, 32 genes had RNA edits. All editing caused amino acid changes, of which the S→L transformed form occurred most frequently. Among all genes in which editing occurred, the *ndhB* had the most abundant editing sites (up to 15). In addition, analysis of the codon positions where editing occurred revealed that editing occurred at the first and second codon positions but not at the third.

### 3.4. Genomic Comparison with Other Species in Machilus

**Figure 4:**
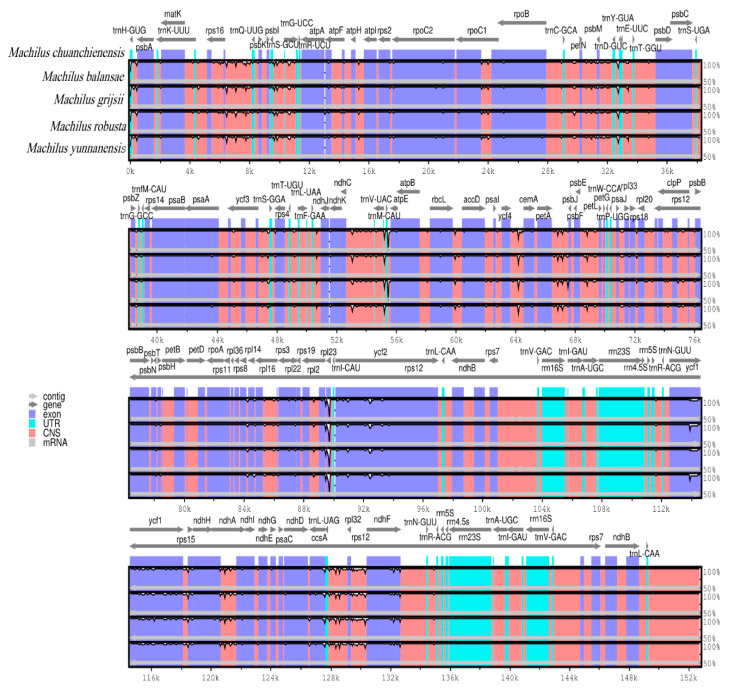
Variations in the chloroplast genome sequences of *M. chuanchienensis* and four related species. The gray arrows indicate the location and orientation of the genes, and the white part indicates the variations between the sequences. CNS represents conservative non-coding sequence.

The *M. chuanchienensis* chloroplast genome (NC_062133) was used as a reference for the global comparison via the online genome comparison tool mVISTA [36], and a comparison of the chloroplast genome sequences of its four related species revealed that these sequences were little changed overall, except for individual sequences in certain regions. First, compared to the conservative protein-coding regions, the intergenic spacer regions in the genomic sequences of the five chloroplasts were significantly more variable. There was little alteration in the rRNA genes, which were largely conserved. The rRNA genes were highly conserved with little variation. The genes in coding regions, such as *ndhF*, *ycf1*, *ccsA*, *rps15*, *rpl23*, and *ycf2*, were highly variable and visualized with large differences in peak maps (Figure 4). In the intergenic regions, *psbA*-*trnH*-*GUG*, *trnQ*-*UUG*-*rps16*, *trnD*-*GUC*-*trnY*-*GUA*, *ndhK*-*atpB*, *ycf4*-*cemA*, *rbcL*-*accD*, *psbE*-*petL*, *petA*-*psbJ*, *ndhH*-*ndhA*, *rpl32*-*trnL*-*UAG*, and *rpl32*-*ndhF* had a higher divergence. In addition, compared to the IR region, the LSC and SSC regions had much higher genetic variability.

The chloroplast genes of *M. chuanchienensis* with the other four species in *Machilus* were compared using the nonsynonymous (Ka) and synonymous (Ks) replacement rates to determine whether selection had taken place. (Table S5). We calculated the Ka/Ks values of 76 common protein-coding genes, and those with a Ka or Ks value of 0 were not included in the statistics. The results showed that the Ka/Ks ratio of *M. chuanchienensis* to *M. balansae* was between 0.0488 (*rpoC1*) and 1 (*ndhB*); *M. chuanchienensis* to *M. grijsii* was between 0.0583 (*rpoC1*) and 2.6552 (*matK*); *M. chuanchienensis* to *M. robusta* was between 0.015 (*psaA*) and 1.3509 (*matK*); and *M. chuanchienensis* to *Machilus yunnanensis* was between 0.0583 (*rpoC1*) and 2.6552 (*matK*). The two *ndhA* and *matK* exceeded 1.0, whereas most genes were below 1.0, suggesting that most genes have undergone purifying selection.

**Figure 5:**
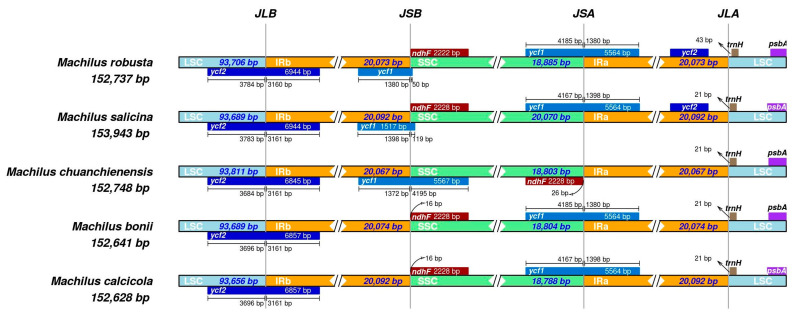
Comparison of the junction regions between LSC and IRb (JLB), LSC and IRa (JLA), IRa/IRb and SSC (JSA and JSB) among the chloroplast genome of five *Machilus* species. The LSC, IRa/IRb, and SSC regions are represented in light blue, orange, and green, respectively, in this map, while other colors represent genes on both sides of the boundary. The number above represents the distance in base pair between the ends of the genes and the boundary site (the distances in this graph are not plotted proportionally).

The expansion and contraction of IR boundaries among five *Machilus* species (Figure 5), including *M. robusta*, *M. salicina*, *M. chuanchienensis*, *M. bonil*, and *M. calcicola*, showed that there were fewer differences in these five species’ IR regions’ chloroplast genome lengths (20,067–20,092 bp) and that the IR/SC boundaries were distributed with *ycf2*, *ycf1*, *ndhF*, and *trnH* genes. In addition, the chloroplast genome of *M. chuanchienensis* had some noticeable structural differences compared with the other four species. For instance, the *ndhF* gene was located in the SSC region but not at the same site as other species, and *ycf1* was not at the JSA site.

**Figure 6:**
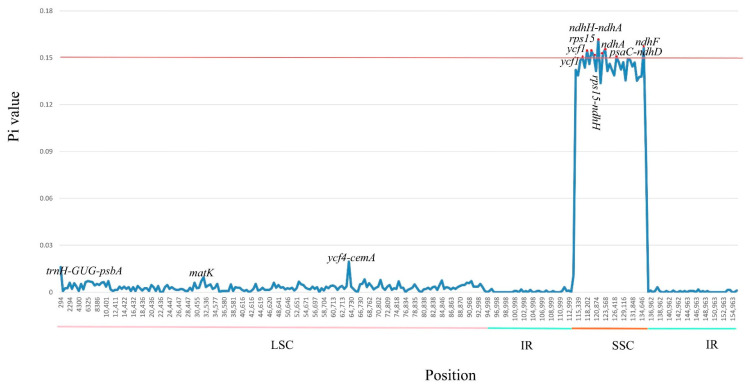
Comparison of the nucleotide variability (Pi) values among the whole chloroplast genome of five *Machilus* species. X-axis: positions of the midpoints of a window, Y-axis: nucleotide diversity in each window.

To examine levels of sequence divergence of the *M. balansae*, *M. grijsii*, *M. robusta*, *M. chuanchienensis*, and *M. yunnanensis*, we calculated the values of nucleotide variability (Pi). The Pi values within 500 bp among the five genomes vary from 0 to 0.1618 with a mean of 0.0176. In addition, we identified seven hypervariable loci (Pi > 0.15), which are *ndhH*-*ndhA* (0.1618), *ndhF* (0.1564), *ndhA* (0.1554, 0.1525) *ycf1* (0.1545, 0.1502), *rps15* (0.1543), *rps15-ndhH* (0.1511), *psaC*-*ndhD* (0.1504). They are all in the SSC region (Figure 6), indicating that the SSC regions were much more divergent than IR and LSC regions and that the IR regions were highly conserved, consistent with the above analysis.

### 3.5. Phylogenetic Analyses

**Figure 7:**
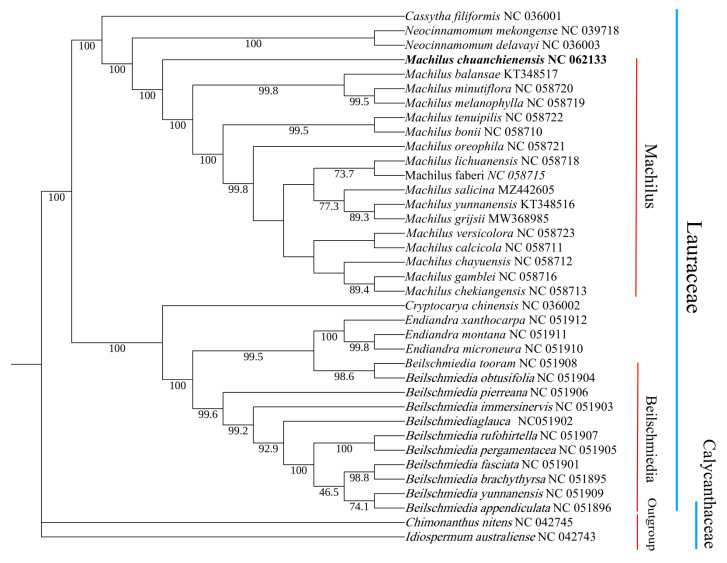
Maximum-likelihood phylogenetic tree of *M. chuanchienensis* based on the whole chloroplast genomes of 36 previously reported species (Numbers on the nodes are bootstrap values from 1000 replicates).

In order to clarify the phylogenetic status and evolutionary relationship of *M. chuanchienensis* in Lauraceae, the whole chloroplast genome sequences of 36 reported species were selected to construct the ML phylogenetic tree with two Calycanthaceae species as outgroups. According to the findings, two distinct groupings can be made up of all Lauraceae species: the genera *Machilus*, *Neocinnamomum*, and *Cassytha* clustered into one group, while *Cryptocarya*, *Endiandra*, and *Beilschmiedia* clustered into another. *M. chuanchienensis* was the first to be separated from the sister clade of the genus *Machilus* with a 100% bootstrap value (Figure 7).

## 4. Discussion

The published lengths of the cp genomes of the genus *Machilus* ranged from 152,621 to 153,943 bp [19,45,46]. In comparison, the total length of the whole cp sequence of *M. chuanchienensis* assembled in this study was 152,748 bp, indicating that its cp genome size was in line with the traits of the *Machilus* species. Furthermore, the cp genome of the *Machilus* was found to be highly conserved when the *M. chuanchienensis* and the reported *Machilus* species in the Lauraceae family were compared. Additionally, it has been reported that the genus *Machilus* has 113–128 total cp genes. In this study, *M. chuanchienensis* cp genome was annotated to 126 genes (82 protein-coding genes, 36 tRNA genes, and eight rRNA genes). The cp genome GC content of *Machilus* species was similar at 39.15% to 39.16%. However, the GC content in the LSC region (37.93% to 33.95%) and SSC region (33.90% to 34.04%) was significantly lower than that in the IR region (41.43%), probably because the eight rRNA genes with higher GC content were distributed in the IR region in this study, which is similar to previous studies on other angiosperms cp genomes [47,48]. Furthermore, similar to other studies [49,50], three genes (*clpP*, *rps12*, and *ycf3*) in the cp genome of *Machilus* contained two intron regions. *ClpP* encodes the Clp proteolytic enzyme subunit, whose function is primarily responsible for the degradation of abnormal proteins and is associated with maintaining the normal metabolism of chloroplasts [51]. Moreover, Boudreau et al. [52] showed that the gene *ycf3* interacts with the PSI subunits at the post-translational level [53] and is required for the accumulation of the photosystem I (PSI) complex. Therefore, more research on these genes is required.

The variation in SSR copy number in chloroplasts is an essential molecular marker with a more significant taxonomic distance than nuclear and mitochondrial microsatellites. It has various applications in plant population genetics, polymorphism, and evolutionary studies [54,55]. A total of 92 simple sequence repeats and 31 long repeats were obtained through in-line software analysis, which can provide candidate molecular markers for related studies such as genetic diversity and conservation genetics of *M. chuanchienensis*. According to studies, *M. chuanchienensis*’ cp genome’s SSRs were relatively abundant in polyadenine (poly-A) or polythymine (poly-T) repeats and seldom contained tandem guanine (G) or cytosine (C), which was similar to other plant cp genomes that have been reported [32,56,57]. According to the results of this study, mononucleotide repeats (72.83%) were the most repeated, which was in line with previous studies [58]. Additionally, a high percentage of forward repetitions (41.94%) were discovered among the four types of repeats, consistent with other studies that demonstrated forward repeats to be the most prevalent [59].

Different species’ genomes exhibit varying relative synonymous codon usage (RSCU). There are biases in codon usage, which can provide critical information for studying species evolution [60]. In addition, codons play a role in vector design for chloroplast genetic engineering and are generally optimized first for vector design [61]. The main reason for codon preference selection is that some preferred codons are more efficient in translation [62]. This study, 23,598 codons were found in all protein-coding genes in the *M. chuanchienensis* cp genome. The most used codons were AAA, GAA, AUU, and AAU, similar to the previous studies in other angiosperms [31,63,64].

The most conserved portion of the cp genome is the IR region, as is widely known. The IR, LSC, and SSC regions’ growth and contraction are frequent evolutionary occurrences and the main factor influencing variations in the length of the cp genome [65,66,67]. This study showed that the length of the IR regions of the cp genome among the related species was less different (20,067–20,092 bp). In addition, studies have shown that repetitive sequences are the leading cause of fragment duplication, deletion, and rearrangement of the cp genome [68]. The *ycf1* and *ndhF* genes are significantly rearranged in the *M. chuanchienensis* compared with the *M. robusta*, *M. salicina*, *M. bonil*, and *M. calcicole*. It was also found in *Dendrobium thyrsiflorum species* [69], and it might be a variant in the cp genome’s boundary area that changed the structure of the cp gene, which may be a variation in the boundary region of the cp genome that led to changes in chloroplast gene structure [70]. The analysis results based on the mVISTA software showed that the noncoding regions occurred at a relatively higher level of divergence than the coding regions [63]. We identified that in the intergenic regions, *psbA*-*trnH*-*GUG*, *trnQ-UUG*-*rps16*, *trnD*-*GUC-trnY*-*GUA*, *ndhK-atpB*, *rbcL*-*accD*, *ycf4*-*cemA*, *petA*-*psbJ*, *psbE*-*petL*, *ndhH*-*ndhA*, *rpl32*-*trnL*-*UAG*, and *rpl32*-*ndhF* had a higher divergence. At the species level, these areas could undergo faster replacement. Understanding and mastering these mutation hotspots will make it easier to comprehend the evolutionary characteristics of the genus *Machilus* cp genome and allow the design of molecular markers based on these sequence fragments to identify molecular DNA barcode screening in the genus [71].

The Ka/Ks ratio mainly reflects the selection pressure of protein-coding genes, which is a meaningful way to detect whether protein-coding sequences have evolved. In this study, the vast majority of Ka/Ks ratios were less than 1% (97%), indicating that most of the genes in the Lauraceae family undergo purification selection, which was consistent with the results of previous studies in the Lauraceae family [72]. Moreover, the results showed that two Ka/Ks ratios were more significant than 1 (*matK*, *ndhA*), indicating that they were significantly positively selected. The *ndhA* belongs to the NADH dehydrogenase subunit maturase gene. The chloroplast NDH monomer sensitive to bright light may have undergone dramatic changes, resulting in the development of new anti-stress functions, and positive selection has existed in the study of *Quercus* [73]. The *matK* gene is located in an intron between two exons with a highly conserved chloroplast lysine tRNA gene (*trnK*), with a sequence length of about 1500 bp. It is a single-copy coding gene encoding a mature enzyme (maturase) involved in the cleavage of type II introns in RNA transcripts [74]. *matK* is often used as a phylogenetic signal to address evolutionary relationships due to its high amino acid replacement rates and nucleotide [74]. However, a positive selection site in *matK* of *Machilus* suggests that this positive selection corrects beneficial variation of *Machilus*, and positive selection has existed in the study of *Chrysosplenium* [75]. Unfortunately, there are few Ka/ka analyses of Lauraceae. Previous studies found that only two genes, *rpl16* and *ycf2*, had Ka/Ks values greater than 1 through the analysis of nine Lauraceae species [72]. Sequence mutational hotspots, also known as hyper-variable regions, provide a reference for designing accurate and efficient molecular markers and species barcodes [76]. The analysis of nucleotide diversity value calculated by the DnaSP software revealed that the SSC regions have high variability, which has also been found in other Lauraceae [77]. It also proves SSCs generally have a higher nucleotide replacement rate than IRS in land plants [78]. The *ndhA* requires our special attention and in-depth study, which has both a high Ka/Ks value and a Pi value. These may indicate that the *ndhA* has undergone a considerable mutation, which is crucial for the evolutionary process of the *Machilus* species.

The plant cp genome is second only to the nuclear genome and has much genetic information [79]. Therefore, whole-genome sequencing technology provides a new platform and idea to study the evolution system of medicinal plants [80]. We employed next-generation sequencing technologies for our sequencing. It is less expensive than first-generation sequencing technologies and does not need cloning, DNA sequence amplification, or strand termination, thus increasing sequencing speed and throughput [81]. Currently, there are two main methods to obtain cp genomes: one is to rely on traditional methods, first isolate chloroplasts, then extract chloroplast DNA and send it for sequencing, and finally assemble and splice to obtain cp genomes. Since the content of chloroplasts in plants is already small, it is not easy to completely separate chloroplast DNA and nuclear genomic DNA. This method is challenging to operate and takes a long time, so it is limited in terms of application. The second is the more commonly used method we adopt, the total DNA of the extract species is sequenced with high throughput, the chloroplast sequence of the species’ close relatives is found as the reference sequence, and the results of the sequencing are compared to find the reads belonging to the chloroplast, and finally assembled. This method first breaks the shackles of traditional methods, eliminates the step of isolating chloroplasts, reduces the time and expense of the experiment, and improves the accuracy of the experiment. In this experiment, we used the second method to obtain the complete cp genome of *M. chuanchienensis* and perform a phylogenetic analysis. As in previous studies [82], the *Machilus* genera came together in this study. The results here showed that all Lauraceae species could be divided into two broad groups, genera *Machilus*, *Neocinnamomum*, and *Cassytha* clustered into one group; *Cryptocarya*, *Endiandra*, and *Beilschmiedia* clustered into another. The phylogenetic relationship of the *Machilus* genera obtained in this study was consistent with the results obtained by Wu et al. [45]. The results of this study will contribute to the subsequent phylogenetic studies and species identification of *Machilus* genera.

## 5. Conclusions

This study yielded the first complete sequence of the *M. chuanchienensis* cp genome. A comparative analysis of the cp genomes in five *Machilus* species was performed. The findings showed that all genomes of the species mentioned in this study exhibited a degree of relative conservation in terms of their content, gene order, and structure. However, the *ycf1* and *ndhF* in the *M. chuanchienensis* were significantly rearranged. The position of *M. chuanchienensis* inside a phylogenetic tree created using the whole cp genome was evident. In addition, 92 SSRs that can be employed in breeding, population genetics, and evolutionary research were found. These findings may offer a clear foundation for the phylogenetic relationships of the *M. chuanchienensis* and provide essential data for exploring and utilizing tea-like species resources.

## Figures and Tables

**Table 1:** Genes found in the assembled *M. chuanchienensis* chloroplast genome.

Category of Genes	Group of Genes	Name of Genes
**RNA genes**	Transfer RNA	*trnH-GUG*, *trnK-UUU ^b^*, *trnQ-UUG*, *trnS-GCU*, *trnG-UCC ^b^*, *trnR-UCU*, *trnC-GCA*, *trnD-GUC*, *trnY-GUA*, *trnE-UUC*, *trnT-GGU*, *trnS-UGA*, *trnG-GCC*, *trnfM-CAU*, *trnS-GGA*, *trnT-UGU*, *trnL-UAA ^b^*, *trnF-GAA*, *trnV-UAC ^b^*, *trnM-CAU*, *trnW-CCA*, *trnP-UGG*, *trnI-CAU*, *trnL-CAA* (×2), *trnV-GAC* (×2), *trnI-GAU ^b^* (×2)*, trnA-UGC ^b^* (×2), *trnR-ACG* (×2), *trnN-GUU* (×2), *trnL-UAG*
	Ribosomal RNA	*rrn23* (×2), *rrn16* (×2), *rrn5* (×2), *rrn4.5*(×2)
**Transcription and translation related genes**	DNA dependent RNA polymerase	*rpoA*, *rpoB*, *rpoC1 ^b^*, *rpoC2*
	Large subunit of ribosome	*rpl2 ^b^*, *rpl14*, *rpl16 ^b^*, *rpl20*, *rpl22*, *rpl23*, *rpl32*, *rpl33*, *rpl36*
	Small subunit of ribosome	*rps2*, *rps3*, *rps4*, *rps7* (×2), *rps8*, *rps11*, *rps12 ^ac^* (×2), *rps14*, *rps15*, *rps16 ^b^*, *rps18*, *rps19*
**Photosynthesis-related genes**	ATP synthase	*atpA*, *atpB*, *atpE*, *atpF ^b^*, *atpH*, *atpI*
	Photosystem I	*psaA*, *psaB*, *psaC*, *psaI*, *psaJ*
	Photosystem II	*psbA*, *psbB*, *psbC*, *psbD*, *psbE*, *psbF*, *psbI*, *psbJ*, *psbK*, *psbM*, *psbN*, *psbT*, *psbZ*
	Cytochrome b/f complex	*petA*, *petB ^b^*, *petD ^b^*, *petG*, *petL*, *petN*
	Large subunit of rubisco	*rbcL*
	NADH dehydrogenase	*ndhA ^b^*, *ndhB ^b^* (×2), *ndhC*, *ndhD*, *ndhE*, *ndhF*, *ndhG*, *ndhH*, *ndhI*, *ndhJ*, *ndhK*
**Other genes**	Translational initiation factor	*infA*
	Acetyl-CoA carboxylase	*accD*
	Maturase	*matK*
	Protease	*clpP ^a^*
	Envelop membrane protein	*cemA*
	c-type cytochrom synthesis gene	*ccsA*
**Unknown function**	Conserved open reading frames	*ycf1*, *ycf2*, *ycf3 ^a^*, *ycf4*

Note: *^a^* Gene containing two introns; *^b^* gene containing a single intron; *^c^* gene divided into two independent transcription units; (×2) gene with two copies.

**Table 2:** Location and length of genes containing introns in *M. chuanchienensis* chloroplast.

Gene	Location	ExonI(bp)	IntronI(bp)	ExonII (bp)	IntronII (bp)	ExonIII (bp)
*ndhA ^+^*	SSC	553	1119	539		
*atpF ^−^*	LSC	145	725	410		
*clpP ^−^*	LSC	71	776	294	652	244
*petB ^+^*	LSC	6	790	642		
*petD ^+^*	LSC	8	716	475		
*rpoC1 ^−^*	LSC	453	718	1620		
*rps16 ^−^*	LSC	40	852	230		
*trnG-UCC ^+^*	LSC	23	765	48		
*rpl16 ^−^*	LSC	9	976	396		
*rpl2 ^−^*	LSC	392	672	430		
*trnK-UUU ^−^*	LSC	37	2507	35		
*trnL-UAA ^+^*	LSC	35	479	50		
*rps12 ^#^*	LSC	114	—	232	536	26
*trnV-UAC^-^*	LSC	39	589	35		
*ycf3 ^−^*	LSC	124	734	230	730	153
*ndhB ^+^*	IR	721	702	758		
*ndhB ^−^*	IR	721	702	758		
*trnA-UGC ^+^*	IR	38	798	35		
*trnA-UGC ^−^*	IR	38	798	35		
*trnI-GAU ^+^*	IR	37	945	35		
*trnI-GAU ^−^*	IR	37	945	35		

Note: ^+^ Exon is transcribed counterclockwise in Figure 1; *^−^* Exon is transcribed clockwise in Figure 1; —spliceosomal intron; ^#^ rps12 is a trans-spliced gene with the 5′ end located in the large single copy (LSC) region; it is duplicated in the 3′ end in the IR regions.

## Data Availability

The data that support the findings of this study are openly available in GeneBank at https://www.ncbi.nlm.nih.gov/ (accessed on 24 January 2022). The complete chloroplast genome has been deposited in GeneBank with accession number OL685165. The associated BioProject, SRA, and Bio-Sample numbers are PRJNA797553, SRS11758196, and SAMN25010768, respectively.

## Supplementary Materials

The following supporting information can be downloaded at: https://www.mdpi.com/article/10.3390/genes13122402/s1, Table S1: Number of different SSRs types in *M. chuanchienensis*; Table S2: Long repeat sequences in the *M. chuanchienensis* chloroplast genome; Table S3: Codon usage of *M. chuanchienensis* chloroplast genome (used Universal Genetic code); Table S4: Putative RNA editing sites in the *M. chuanchienensis* chloroplast genes; Table S5: The nonsynonymous (Ka) and synonymous (Ks) replacement rates of the *M. chuanchienensis* chloroplast genome.
